# The importance of standardizing criteria for PGT-A interpretation of blastocysts analyzed by next-generation sequencing

**DOI:** 10.5935/1518-0557.20230011

**Published:** 2023

**Authors:** Anna Calull Bagó, María Teresa Izaguirre Hernández, Patricia Cancino Villarreal, Claudia González Ortega, Antonio Martín Gutiérrez Gutiérrez

**Affiliations:** 1 Institute of Sciences in Human Reproduction “Vida”, Guanajuato, Mexico

**Keywords:** preimplantation genetic testing, mosaicism, blastocyst, NGS.

## Abstract

**Objective:**

To compare the preimplantation genetic testing for aneuploidy (PGT-A) results using the three most frequent criteria employed by preimplantation genetic laboratories and evaluate its impact on the number of euploid embryos available for transfer.

**Methods:**

Retrospective and descriptive study including patients who underwent PGT-A between January 2018 and December 2020. Five hundred and nine PGT-A cycles and 2,079 blastocysts were analyzed by next-generation sequencing (NGS). We re-assigned the diagnosis of all blastocysts using three different criteria: strict (mosaicism thresholds from 20% to 80%), standard (from 30% to 70%) and excluding (mosaicism is not reported). We compared the euploid, aneuploid and mosaic embryos obtained in each criteria used.

**Results:**

We observed PGT-A results discrepancies in 32.5% (165/509) of the cycles when the three different criteria were applied. The standard and excluding criteria showed 92 more euploid embryos (875/2,079) compared to the strict criteria (783/2,079). Evaluating the PGT-A results per cycle with the strict, standard and excluding criteria, the euploidy rates were 34.0%, 38.4% and 38.4% (*p*<0.001); aneuploidy rates were 59.0%, 55.8% and 61.6% (*p*<0.001) and mosaic rates were 7.0% and 5.8% (*p*<0.047), respectively. The mean number of euploid blastocysts available for transfer was 1.54±1.67 with the strict criteria, while the possibility to obtain an euploid embryo was higher if the standard or the excluding criteria were used 1.72±1.78 (*p*<0.001).

**Conclusions:**

This study highlights the importance of standardizing the criteria used for the interpretation of PGT-A blastocysts. We observed significant differences on PGT-A results associated solely to the criteria used.

## INTRODUCTION

Aneuploidy is considered one of the main factors that can adversely affect in vitro fertilization (IVF) outcomes. During the early human embryo development, a high prevalence of aneuploidy exists, leading to early pregnancy loss and failure of IVF treatments. Moreover, aneuploidy is associated with maternal age reaching up to 80% or greater of aneuploid embryos in patients older than 40 years (Franasiak *et al*., 2014a). Most of these chromosomal aberrations are going to cause embryo arrest, implantation failure or early miscarriage, if the embryo implants (Hassold & Hunt, 2001; Macklon *et al*., 2002; Nagaoka *et al*., 2012; Kort *et al*., 2016; Ottolini *et al*., 2017). This is because each chromosome carries hundreds of genes, and the addition or loss of one or more chromosomes affects the cellular genetic balance. Nevertheless, some of them like trisomy 13, 18, 21 and aberrations in sex chromosomes are compatible with embryo viability and fetal development resulting in live births with a chromosomopathy. Preimplantation genetic testing for aneuploidy (PGT-A) is a modern cytogenetic tool introduced into clinical assisted reproduction technology (ART) practices that allows the detection of chromosomal alterations in embryos produced by IVF in order to select the euploid ones for uterine transfer. In this manner, we can improve the implantation rates, reduce the time to achieve pregnancy, reduce the miscarriage rates and avoid newborns with chromosomal alterations (Scott Jr *et al*., 2013; Harton *et al*., 2013; Simon *et al*., 2018; Munné *et al*., 2019).

Preimplantation genetic testing was carried out for the first time in late 80´s when PCR was used for sex selection on day three embryos form couples at risk for transmitting X-linked recessive disorders (Handyside *et al*., 1990). Since then, several methods have been applied for aneuploidy screening like fluorescence in situ hybridization (FISH), quantitative PCR (qPCR), comparative genomic hybridization array (aCGH) and single nucleotide polymorphism array (SNP array). Nowadays, next-generation sequencing (NGS) is considered the gold standard for PGT-A due to its higher sensitivity and specificity compared to technologies previously used (Friedenthal *et al*., 2018).

Currently, thanks to the advances of aneuploidy screening techniques and the biopsy of multiple trophectoderm (TE) cells of the blastocysts, we cannot just discriminate between euploid and aneuploid embryos, but we also can detect mosaic embryos. Mosaicism is defined as the presence of two or more chromosomally distinct cell lines in a given embryo or organism. The incidence reported in the literature of this phenomenon is highly variable ranging from ≈ 3 to 40% (Johnson *et al*., 2010; Capalbo *et al*., 2013; Sachdev *et al*., 2017) but is reduced from ≈ 2% to 17% (Ruttanajit *et al*., 2016; Katz-Jaffe *et al*., 2017; Stankewicz *et al*., 2017; Nakhuda *et al*., 2018) in studies based on NGS analysis of a single TE biopsy.

The possibility of detecting mosaic embryos by PGT-A, opened a new window which we were not ready to look through. Some recommendations and committee opinions have been published to help clinicians with the management of mosaic results (Sachdev *et al*., 2017; Cram *et al*., 2019; Practice Committee and Genetic Counseling Professional Group of the American Society for Reproductive Medicine, 2020; Chen *et al*., 2021). Despite a mosaic result is not a normal result, it has been demonstrated that some of these embryos are able to achieve viable pregnancies (Greco *et al*., 2015; Lledó *et al*., 2017; Fragouli *et al*., 2017; Munné *et al*., 2017; 2020; Spinella *et al*., 2018; Zhang *et al*., 2019; Victor *et al*., 2019a; Lin *et al*., 2020; Viotti *et al*., 2021; Capalbo *et al*., 2021). Mosaicism is a post-zygotic event, therefore, it is not age-related (Escudero *et al*., 2016; Calull-Bagó *et al*., 2020) but there are many factors that can impact the mosaicism rate, for example the methodology used for the aneuploidy screening, in vitro culture conditions, the biopsy technique, sampling site of the embryo or bias in the library preparation (Deleye *et al*., 2015; Maxwell *et al*., 2016; Popovic *et al*., 2018; Swain, 2019; Victor *et al*., 2019b; Xiong *et al*., 2021). Nevertheless, one of the main factors that can impact the incidence of mosaicism and the PGT-A results is the lack of standardization in reporting and interpreting mosaic embryos among PGT laboratories.

In 2019, the Preimplantation Genetic Diagnosis International Society (PGDIS) published a position statement where they suggested that embryos ranging from 20% to 80% of mosaicism should be diagnosed as mosaic embryos (Cram *et al*., 2019). Accordingly, those with a level of mosaicism lower than 20% should be diagnosed as euploid embryos and greater than 80% as aneuploid. On the other hand, it has been described that bioinformatic analysis used in NGS is not capable of discriminating low levels of mosaicism from technical artifacts and experimental noise (Capalbo *et al.,* 2017; Treff & Franasiak, 2017). Based on these facts, some authors such as Simón & Rubio (2017), after performing their own validation, preferred to apply a less strict criteria and use a range from 30% to 70% for mosaicism diagnosis, consequently avoiding false positives (Simón & Rubio, 2017; Rubio *et al*., 2019). Finally, there is one last group of authors who defend that chromosome copy number value (CNV) provided by NGS is insufficient to predict a real embryonic mosaicism. So, they mention that PGT-A laboratories should limit their results to euploid when the degree of mosaicism detected is below 30% and aneuploid when above 30% (Lawrenz *et al*., 2019; Treff & Marin, 2021).

To date, there has not been a study that compares the different criteria used in the diagnosis of PGT-A embryos. The aim of this study is to compare the PGT-A results using the three most frequent criteria employed by preimplantation genetic laboratories and evaluate its impact on the number of embryos available for transfer.

## MATERIAL AND METHODS

This is a retrospective and descriptive study including patients who underwent PGT-A in multiple clinics from January 2018 to December 2020. Written informed consent was obtained from all the patients. In total, 509 PGT-A cycles and 2,079 blastocysts were analyzed during this period.

To evaluate the impact of different criteria used for the interpretation of PGT-A embryos, the number of euploid blastocysts available for transfer was established as the primary outcome. The secondary outcome was the proportion of PGT-A cycles with discordant results between different criteria. We also evaluate the mosaicism rate from our blastocyst cohort and chromosome-specific distribution of mosaicism.

During the study period, infertile patients attending in vitro fertilization (IVF) clinics were offered the PGT-A; mainly in those patients with advanced maternal age (≥ 35 years at the day of oocyte retrieval), recurrent pregnancy loss history or recurrent implantation failure but also patients with severe male factor, patients who wanted an euploid embryo transfer (regardless of their age) and patients with desire of family balance. All patients enrolled in the study performed one or more IVF and intracytoplasmic sperm injection (ICSI) cycle following the controlled ovarian hyperstimulation protocol that their physician thought best suited to them. All embryos were biopsied on day 5, 6 or 7 after fertilization depending on their expansion grade and morphological quality and 5 to 10 cells were obtained from the trophectoderm. The biopsies were placed into 2.5 µl of PBS 1X buffer (Gibco^TM^, USA), frozen at -20°C and shipped to our laboratory. Subsequently, blastocysts were vitrified pending for the PGT-A results.

All trophectoderm biopsies were submitted to our preimplantation genetics laboratory (Vida Genetics, Mexico) for aneuploidy screening analysis. The samples were analyzed by NGS following the manufacturer instructions (Ion ReproSeq^TM^ PGS Kit, Thermo Fisher Scientific, USA). Briefly, the biopsied cells were prepared for DNA extraction and whole genome amplification (WGA) using the Ion SingleSeq^TM^ Kit (Thermo Fisher Scientific, USA). Following the WGA, each sample was labeled with a unique barcode (known DNA sequences) which allows that samples can be mixed during the sequencing procedure and afterwards matched with their original embryo. Then, an isothermal amplification was performed on the DNA obtained from the WGA and the sequencing primer and polymerase were incorporated to proceed with the final sequencing step. The NGS procedure was completed using the Ion Personal Genome Machine sequencer (Thermo Fisher Scientific, USA) and the resulting sequences were aligned with a reference human genome (hg19). The data analysis was performed with Ion Reporter Software v.5.16 (Thermo Fisher Scientific, USA) which provides the CNV for each chromosome and specific regions of each chromosome, which is expected to be two (one for male sex chromosomes).

**Figure 1:**
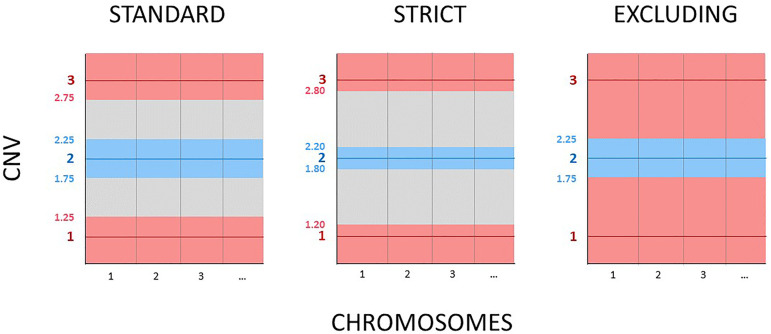
Simplified comparison of the thresholds used by the three criteria evaluated in this study for the interpretation of PGT-A blastocysts. Blue area: euploid range, grey area: mosaic range, red area: aneuploid range. CNV= copy number value.

For this study, we re-assigned the diagnosis of the 2,079 blastocysts processed in our laboratory from January 2018 to December 2020 using the three criteria most frequently employed by preimplantation genetic laboratories. We named them as “strict”, “standard” and “excluding” criteria. For the strict criteria, we applied the thresholds from 20% to 80% which means that chromosomes with CNV between 1.8 and 2.2 were considered as normal (euploid embryos), chromosomes showing CNV below 1.2 or above 2.8 were considered as monosomy or trisomy respectively, or deletions or duplications in case of segmental alterations (aneuploid embryos). Everything else outside these ranges was considered as mosaic (CNV from 1.2 to 1.8 and 2.2 to 2.8) (Cram *et al*., 2019). For standard criteria, we took the thresholds from 30% to 70%. Embryos were diagnosed as euploid when CNV were between 1.7 and 2.3, aneuploid when CNV were below 1.3 or above 2.7 and mosaic when CNV fell outside these ranges (from 1.3 to 1.7 and 2.3 to 2.7) (Simón & Rubio, 2017; Rubio *et al*., 2019). Finally, for the excluding criteria, the CNV range considered for an euploid diagnosis was the same as standard criteria (between 1.7 and 2.3) but in this case, all embryos with CNV outside this threshold were considered aneuploid (Lawrenz *et al*., 2019; Treff & Marin, 2021) (Figure 1). In the excluding criteria, there is not a mosaic category. In all three criteria, we reported an embryo as aneuploid when we found more than one chromosome classified as mosaic, as well as when the embryo was aneuploid/aneuploid mosaic.

Statistical analysis was carried out using IBM SPSS Statistics v.24 (IBM, USA). A *p* value of <0.05 was considered to identify statistical significance for all statistical tests. ANOVA test was used to assess general differences between the results from the three criteria applied while Bonferroni test was used for multiple comparisons.

## RESULTS

The study includes the data analysis from 2,079 blastocysts with a valid diagnosis obtained from 509 PGT-A cycles. Basal and cycle characteristics of the patients participating in this study are summarized in Table 1. The mean female age of the patients that performed PGT-A cycles with autologous oocytes (459, 90.2%) was 37.3±4.8 (range 20 - 49) and the mean age of the donors from the oocyte donation PGT-A cycles (50, 9.8%) was 24.0±3.2 (range 18 - 35). The most common indication was advanced maternal age (47.0%), followed by infertile patients which elect to perform the PGT-A to ensure an euploid embryo transfer (14.9%). A similar percentage was observed in the PGT-A cycles for family balancing (13.9%) followed by recurrent implantation failure (9.4%) and recurrent pregnancy loss (8.1%). Severe male factor (3.9%) as sole indication represented a minority of cases. Moreover, there were 14 PGT-A cycles (2.8%) with indications other than those mentioned above including a previous genetically abnormal pregnancy, karyotype with altered sex chromosomes (XYY, XXX) or desire to postpone pregnancy having euploid embryos preserved.

**Table 1:** Basal and cycle characteristics of the patients included in the study.

Characteristics	Values
PGT-A cycles, n	509
With autologous oocytes, n (%)	459 (90.2)
With oocyte donation, n (%)	50 (9.8)
Mean patient’s age ± SD (range)	37.3±4.8 (20 - 49)
Mean donor’s age ± SD (range)	24.0±3.2 (18 - 35)
PGT-A indication Advanced maternal age, n (%) Elective, n (%) Family balancing, n (%) Recurrent implantation failure, n (%) Recurrent pregnancy loss, n (%) Severe male factor, n (%) Other indications, n (%)	239 (47.0) 76 (14.9) 71 (13.9) 48 (9.4) 41 (8.1) 20 (3.9) 14 (2.8)
Blastocysts analyzed, n	2,079
Mean number of blastocysts analyzed per cycle ± SD	4.1±2.5

From the 2,079 blastocysts analyzed, 1,554 (74.7%) were biopsied on day 5, 489 (23.5%) on day 6 and 36 (1.7%) on day 7 post ICSI. The mean number of blastocysts analyzed per cycle was 4.1±2.5. The overall percentage of euploid, aneuploid and mosaic embryos applying the strict criteria were 37.7% (783), 54.5% (1,134) and 7.8% (162), respectively. Analyzing the same embryos with the standard criteria, the percentage of euploid, aneuploid and mosaic embryos were 42.1% (875), 51.4% (1,069) and 6.5% (135), respectively. Finally, using the excluding criteria, the percentages obtained were as follows: 42.1% (875) of euploid embryos and 57.9% (1,204) of aneuploid embryos. As mentioned before, the excluding criteria does not consider a mosaic category (Table 2).

**Table 2:** Percentage of euploid, aneuploid and mosaic blastocysts depending on the criteria used for their analysis.

PGT-A result	Strict	Standard	Excluding
Euploid, n (%)	783 (37.7%)	875 (42.1%)	875 (42.1%)
Aneuploid, n (%)	1,134 (54.5%)	1,069 (51.4%)	1,204 (57.9%)
Mosaic, n (%)	162 (7.8%)	135 (6.5%)	0 (0%)

Strict criteria: mosaicism thresholds from 20% to 80%; standard criteria: mosaicism thresholds from 30% to 70%; excluding criteria: mosaicism not reported.

The standard criteria showed 92 more euploid embryos compared to the strict criteria, since 65 (70.7%) of them were classified as aneuploid (embryos with more than one chromosome with 25% of mosaicism level) and 27 (29.3%) were classified as mosaic (embryos with only one chromosome with 25% of mosaicism level) following the strict criteria. Regarding the excluding criteria, the same number of euploid embryos as standard criteria was observed since the threshold that defines an euploid embryo is the same on both criteria (mosaicism level <30%). Nevertheless, 135 embryos classified as mosaic according to standard criteria, were now classified as aneuploid (Table 2). Analyzing the mosaicism level from these embryos categorized as aneuploid according to the excluding criteria, we detect that 106 of them (78.5%) presented low degree of mosaicism (<50%) and 29 (21.5%) showed high degree (≥50%). In addition, these 135 blastocysts came from 112 PGT-A cycles from which 22 (19.6%) had no euploid blastocyst for transfer. The proportion of cycles with at least one euploid embryo for transfer was 69.2% (352) with standard and excluding criteria and 65.6% (334) with strict criteria. However, if we consider the low degree mosaic embryos as suitable for transfer, the differences in the percentages of cycles with at least one transferable embryo decrease between criteria, being 70.1% (357) with strict criteria, 72.5% (369) with standard criteria and 69.2% (352) with excluding criteria.

**Figure 2:**
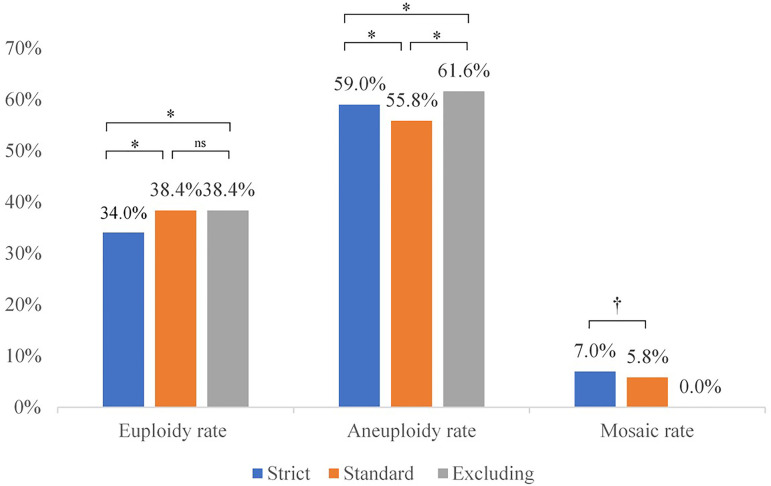
Euploidy, aneuploidy and mosaic rates per PGT-A cycle depending on the diagnostic criteria used. *p<0.001, ^†^*p*<0.05, ns: not significant.

When we look at the PGT-A results per cycle performed, we could observe that there were also differences between the three criteria (Figure 2). The mean euploidy, aneuploidy and mosaic rate obtained with the strict, standard and excluding criteria were as follows: 34.0%, 38.4% and 38.4% (*p*<0.001); 59.0%, 55.8% and 61.6% (*p*<0.001); 7.0% and 5.8% (*p*<0.047) respectively. Applying the Bonferroni test for multiple comparison correction, the differences remained significant except for the euploidy rate between standard and excluding criteria (Figure 2). The number of euploid blastocysts available for transfer would be 1.54±1.67 embryos in average per cycle if the PGT laboratory had used the strict criteria for the diagnostic, while the possibility to obtain an euploid embryo would be higher if the criteria used was the standard or the excluding criteria (1.72±1.78) (*p*<0.001). Taking into account these results, we observed that the percentage of PGT-A cycles with at least one discrepant result applying different criteria was 32.4% (165/509), almost one third of the analyzed cycles, while 67.6% (344) of PGT-A cycles would remain invariable despite the criteria used.

**Figure 3:**
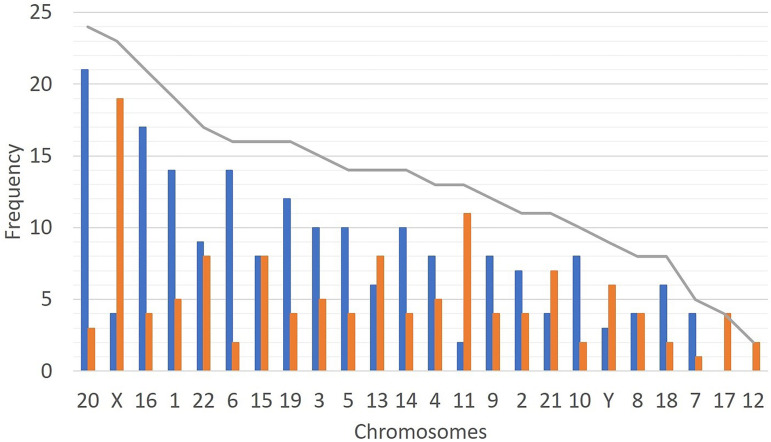
Incidence of chromosomes with intermediate copy number (putative mosaic chromosomes) in the preimplantation blastocysts analyzed (grey line) and the genomic alteration: trisomies (blue bars) and monosomies (orange bars). For the X chromosome, the blue bar represents mosaic XXX embryos and the orange bars mosaic X embryos. For the Y chromosome, the blue bar represents a CNV ≥ 1.25 and orange bar a CNV ≤ 0.75.

Among the 2,079 blastocysts studied, we identified 315 whole chromosomes with intermediate CNV (putative mosaic chromosomes). Assuming we analyzed 95,634 chromosomes, the mean mosaicism rate per chromosome observed was 0.33%. The most common chromosomes with intermediate CNV observed in our blastocyst’s population were chromosomes 20 (24, 7.6%), X (23, 7.3%), 16 (21, 6.7%) and 1 (19, 6.0%). On the other hand, the chromosomes less frequently seen with intermediate CNV were 12 (2, 0.6%), 17 (4, 1.3%) and 7 (5, 1.6%). The prevalence of trisomic mosaic alterations was higher (189, 60.0%) compared with the monosomic alterations (126, 40.0%) (Figure 3).

## DISCUSSION

To our knowledge, this is the first study that compares the PGT-A results obtained by NGS applying three different diagnostic criteria that are used nowadays in PGT laboratories. As we expected, the PGT-A results from the same embryos did vary from one criteria to another.

Currently, the CNV provided by NGS software is the most common method used for predicting mosaicism and establishing the final embryo’s diagnostic. If the CNV from a specific chromosome or chromosomes fall into the mosaic range (between 20% to 80% or 30% to 70% depending on the criteria used), the embryo will be classified as mosaic (or even aneuploid) and it is assumed that the chromosomal abnormality detected originates from post-zygotic errors. Unfortunately, the CNV is far from represent a true mosaicism since other factors can lead to an intermediate copy number. First of all, a genuine mosaicism can only be confirmed performing multiple biopsies including TE cells and inner cell mass (ICM) on the same embryo, however, it is not a feasible procedure in a clinical context. Assuming that a single TE biopsy could be representative of the entire blastocyst, which it is in 93-98% of cases as demonstrated by several studies with a high number of blastocysts analyzed (Huang *et al*., 2017; Lawrenz *et al*., 2019; Victor *et al*., 2019a), the CNV obtained can still be influenced by different factors other than chromosomal constitution of the biopsied TE cells, leading to a false positive mosaicism.

WGA methods can lead to amplification bias which produce unbalances of specific regions of the genome. Consequently, such artifacts can be misinterpreted as mosaic structural aberrations (Sabina & Leamon, 2015). The biopsy itself can also contribute to create false positive mosaicism. The number of laser pulses, the number of biopsied cells (too few or too many), mechanical damage to cells or the introduction of cellular fragments into the PCR tube, all can affect the quality of the embryo profile and the chromosomal CNV (Herrero Grassa *et al*., 2020; Treff & Marin, 2021; Xiong *et al*., 2021; Yelke *et al*., 2021; Zhou *et al*., 2021), although an appropriate biopsy training can mitigate this effect (Capalbo *et al*., 2016). Additionally, poor praxis in terms of sterility during the blastocyst biopsy procedure can also lead to false positive mosaic embryos. Finally, the embryo culture conditions are also considered a source of mosaicism (Swain, 2019). All these factors are quite difficult to control, especially between IVF laboratories and even across different biopsy practitioners from the same laboratory. In consequence, the mosaicism rates reported in the literature vary widely.

In addition to the factors mentioned above, there is one more variable that also affects the mosaicism rates, which is the threshold used by the PGT laboratories for predicting mosaicism (the diagnostic criteria). But contrary to the previous factors, this is much easier to standardize. As we demonstrated in this study, almost one third of PGT-A cycles (32.4%) could have at least one embryo with a different result if other criteria was used; thus, we can assume that one of the main parameters affecting the highly variable mosaicism rates reported in the literature is the diagnostic criteria employed. Therefore, the lack of standardization in reporting presumptive mosaicism makes the comparison between rates pointless.

Some examples of these discrepancies are reviewed below. Nakhuda *et al*. (2018) detected mosaicism as the sole abnormality in 17.5% of samples analyzed using the strict diagnostic criteria. Ruttanajit *et al*. (2016) reported 8.5% of mosaicism also just considering the euploid/aneuploid embryos, but they used a mosaicism threshold from 10% to 90%. Stankewicz *et al*. (2017) reported a 2.4% of mosaicism incidence but they did not mention the diagnostic thresholds used. Nevertheless, they did mention that according to the company policy, any mosaic monosomy or trisomy profile involving chromosomes 13, 16, 18, 21, X and/or Y were interpreted as purely aneuploid. Capalbo *et al*. (2021) reported 29.0% of blastocysts analyzed showing a CNV between 20 - 50%, however, they considered these embryos as euploid and those with CNV higher than 50% as aneuploid. In our study, we observed a 7.8% and 6.5% of mosaic embryos depending on the criteria used. Therefore, even though all these studies used NGS as aneuploidy screening method, the NGS platform has been validated and the incidence of mosaicism was obtained from a single TE biopsy, there are a large variety of additional factors that are conditioning the final mosaicism rate. Our results show that even in the best scenario, where all the possible factors that can impact the PGT-A results are controlled and homogenized, there are still significant differences in the results if the analytical criteria are not standardized.

Attempting to provide a more representative mosaicism rate avoiding the bias introduced by the type of analysis itself, we also reported the prevalence of all chromosomes with intermediate copy number detected in our blastocyst population. For that, we reviewed the NGS results of our 2,079 blastocysts to evaluate the incidence of chromosomes-specific mosaicism. We excluded from this analysis the segmental mosaicism and chaotic embryos (five or more chromosomal abnormalities). The mean mosaicism rate per chromosome observed considering whole-chromosome mosaic alterations was 0.33%. This rate is substantially lower than the one reported by Nakhuda *et al*. (2018) (2.46%). This could be because of the exclusion of chaotic embryos (which sometimes also show mosaic chromosomes) and segmental mosaicism. Besides, their incidence of mosaicism was 17.5%, considerably higher than the incidences observed in our study (7.8% and 6.5%), so we could expect that the mosaicism rate per chromosome was also higher.

The chromosomes most affected by mosaicism per biopsy (20, X, 16 and 1) observed in our blastocyst cohort differ that the ones reported by Nakhuda *et al*. (2018) (21, 22, 2 and 14). We have seen several times a chromosome-specific pattern of distribution of aneuploidies, being reproducible in any blastocyst population, with chromosomes 15, 16, 21 and 22 resulting the most affected (Fragouli *et al*., 2011; Franasiak *et al*., 2014b; McCoy *et al*., 2015; Shahbazi *et al*., 2020). It is believed that short and acrocentric chromosomes are prone to suffer meiotic errors in copy number. Interestingly, it seems that chromosomes affected by mosaicism don’t follow the same pattern. Although the most common chromosomes with intermediate copy number observed in our blastocysts do not match with those reported by Nakhuda, very large chromosomes like 1 and 2 have been detected. These chromosomes typically show low rates of aneuploidy, probably because embryos cannot manage the genetic unbalance which affect its development potential (Rodriguez-Purata *et al*., 2015; Shahbazi *et al*., 2020; Wartosch *et al*., 2021), however, these results probe that blastocysts are tolerant to errors in these chromosomes as long as they are in mosaic. In fact, the only child with probed mosaicism born after the transfer of a known mosaic embryo, presented a mosaic karyotype for chromosome 2 (Kahraman *et al*., 2020).

This study shows that using the standard and the excluding criteria for the diagnosis of PGT-A embryos, improve the likelihood of achieving an euploid embryo compared with the strict criteria (1.72 *vs*. 1.54, respectively, *p*<0.001). On the other hand, the use of the excluding criteria compared with the standard criteria classify a considerable quantity of embryos (135, 6.5%) as clinically unsuitable, while they could be transferred in case of euploid embryos absence after appropriate counseling. We identified that 78.5% of mosaic embryos classified as aneuploid by the excluding criteria presented a low degree of mosaicism. So far, several studies have reported transfers of putative mosaic embryos demonstrating they can achieve viable pregnancies and healthy live births (Greco *et al*., 2015; Lledó *et al*., 2017; Fragouli *et al*., 2017; Munné *et al*., 2017; 2020; Spinella *et al*., 2018; Zhang *et al*., 2019; Victor *et al*., 2019a; Lin *et al*., 2020; Viotti *et al*., 2021; Capalbo *et al*., 2021). To evaluate the reproductive potential of blastocysts with different mosaicism grades and the implication of excluding putative mosaic embryos for transfer, Capalbo *et al. (*2021) analyzed the outcomes following single frozen embryo transfers of 484 euploid blastocysts (<20% of mosaicism), 282 low-degree mosaic blastocysts (20-30%) and 131 moderate-degree mosaic blastocysts (30-50%) sequenced by NGS. Surprisingly, they could not find any differences between these three categories in terms of positive pregnancies (55.8%, 55.0% and 55.7%, respectively), miscarriages (12.0%, 11.0% and 12.7%, respectively) or live birth rates (43.3%, 42.9% and 42.0%, respectively). Besides, all genetic tests derived from the newborns that were analyzed showed fully normal karyotypes (Capalbo *et al*., 2021).

Previous studies also using NGS, showed lower clinical performance of putative mosaic embryos compared to euploid embryos, however, when then compared the reproductive outcomes between euploid and low degree mosaic embryo transfers (defined as <40% or <50%), they found quite similar results. Fragouli *et al.* (2017) observed a significantly poorer performance of mosaic embryos compared with euploid embryos: implantation rates 30.1% *vs*. 55.8% (*p*=0.038), miscarriage rate 55.6% *vs*. 17.2% (*p*=0.036) and ongoing pregnancy rate 15.4% *vs*. 46.2% (*p*=0.03). Nevertheless, they analyzed a small sample of transferred embryos (51 euploid and 44 mosaic blastocysts), did not compare the results between mosaicism degrees, and the thresholds used were poorly described (Fragouli *et al*., 2017). Munné *et al*. (2017), following the strict criteria, evaluated the pregnancy outcomes of 143 mosaic embryo transfers and compared them with 1,045 euploid transfers. Of note, forty-four of these mosaic embryos were included in the previous study performed by Fragouli *et al*. (2017). Mosaic embryos had a lower but not significant implantation rate (53%) compared with euploid embryos (70%), higher fetal loss rate (25% *vs*. 10%, *p*=0.002) and lower ongoing implantation rate (40% *vs*. 63%, *p*=0.006). However, single-chromosome mosaic embryos with low degree of mosaicism (20-40%) had quite similar results compared with euploid embryos: implantation rate 69%, fetal loss rate 19% and ongoing implantation rate of 56% (Munné *et al*., 2017). The results observed by Spinella *et al.* (2018) also follow the same pattern. After the transfer of 78 mosaic blastocysts and 251 euploid blastocysts, they obtained an implantation rate of 38.5% *vs*. 54.6% (*p*=0.02), early abortion rate of 7.8% *vs*. 8.0% (*p*=0.85) and live birth rate of 30.8% *vs*. 46.4% (*p*=0.019), respectively. Nevertheless, embryos with low degree of mosaicism (<50%) resulted in clinical outcomes similar to euploid embryos: implantation rate 48.9% (*p*=0.5), miscarriage rate 6.8% (*p*=0.79) and live birth rate 42.2% (*p*=0.75). They described a mosaicism result as a copy number between 2 and 3 or 2 and 1 (Spinella *et al*., 2018).

On the other hand, Victor *et al.* (2019a) evaluated 100 mosaic embryo transfers and compared the clinical results with 478 euploid transfers. The blastocysts were diagnosed following the strict criteria. As expected, the mosaic group showed significant lower implantation rate compared with the euploid group (38.0% *vs*. 49.6%, *p*=0.0273) and had lower chances of developing a fetal heartbeat (30.0% *vs*. 47.1%, *p*=0.0019). Interestingly, when they compared the outcomes between low and high mosaicism degree applying different thresholds (low: 20-50% or 20-40%; and high: 50-80% or 40-80%), they observed better results in the high mosaicism degree group: implantation rate 40.9% and 38.1% (50-80% and 40-80%, respectively) compared with 35.9% and 36.2% in low degree group (20-50% and 20-40% respectively) (Victor *et al.,* 2019a). Finally, the study with the largest number of transferred mosaic embryos so far is the paper published by Viotti *et al*. (2021). They compared the clinical outcomes of 1,000 mosaic embryos and 5,561 euploid embryos diagnosed by the strict criteria. The euploid group resulted in significantly superior outcomes compared with mosaic group: implantation rate of 57.2% *vs*. 46.5%, ongoing pregnancy/birth rate of 52.3% *vs*. 37.0% and spontaneous miscarriage of 8.6% *vs*. 20.4%. When mosaic embryos were stratified by the level of mosaicism, they found that using 50% or 60% as a cutoff yielded significant differences between low and high mosaicism groups in terms of clinical outcomes. The results stratified by low (<50%) and high (≥50%) degree of mosaicism were as follows: implantation rate 47.8% *vs*. 39.3% and ongoing pregnancy/birth 40.1% *vs*. 28.3%, respectively (Viotti *et al*., 2021).

None of the studies mentioned above describe births with chromosomal alterations. The only birth of a mosaic baby after the transfer of a known mosaic embryo reported in the literature, is the case of a healthy baby girl with a mosaic karyotype for chromosome 2 mentioned previously (Kahraman *et al.,* 2020). A birth of an affected baby who died at 6 weeks was reported from Munné *et al*. (2020) in a study where 10 fully abnormal embryos were transferred. Nine of them do not implant and one end up in a chromosomally abnormal live birth.

There are some limitations to this study. First, as it has been done in other studies, we also interpreted as aneuploid embryos those aneuploid - aneuploid mosaic embryos or those with more than one chromosome with intermediate copy number. This is because we wanted to reproduce the same strategies used in the clinical context, nevertheless, we are aware that this could reduce our mosaicism incidence as well as increment the number of aneuploid embryos. Regarding this study, as we applied the same strategy on the three criteria, it had no impact on our results. Second, as far as we know, we took the tree criteria most commonly used in PGT laboratories to evaluate the importance of the CNV thresholds used. Nevertheless, we are aware that there are “sub-criteria” other than the CNV thresholds which differ from one laboratory to another and can impact the PGT-A results.

In conclusion, this study highlights the importance of standardizing the criteria used for the interpretation of PGT-A blastocysts. We observed significant differences on PGT-A results associated solely to the criteria used, consequently affecting the reproductive success of the patients in treatment. We should consider crucial to request the mosaic rates and thresholds used to the PGT laboratory for mosaic interpretation as long as this standardization is not a fact.
